# Sustainable Cellulose Production from Agro-Industrial Waste: A Comprehensive Review

**DOI:** 10.3390/polym18020153

**Published:** 2026-01-06

**Authors:** Akmaral Darmenbayeva, Reshmy Rajasekharan, Zhanat Idrisheva, Roza Aubakirova, Zukhra Dautova, Gulzhan Abylkassova, Manira Zhamanbayeva, Irina Afanasenkova, Bakytgul Massalimova

**Affiliations:** 1 Department of Chemistry and Chemical Technology, M. Kh. Dulaty Taraz University, Taraz 080000, Kazakhstan; 2 Department of Science and Humanities, Providence College of Engineering, Ala 689122, Kerala, India; reshmypkumar@gmail.com; 3 School of Geosciences, D. Serikbayev East Kazakhstan Technical University, Ust-Kamenogorsk 070003, Kazakhstan; zhidrisheva@edu.ektu.kz (Z.I.); mzhamanbaeva@edu.ektu.kz (M.Z.); 4 Department of Chemistry, S. Amanzholov East Kazakhstan University, Ust-Kamenogorsk 070010, Kazakhstan; roza.aubakirova@bk.ru (R.A.); dautowa@mail.ru (Z.D.); abylkassova@mail.ru (G.A.); iafanasenkova@vku.edu.kz (I.A.); 5 Department of Chemistry and Chemical Technology, M. Kozybayev North Kazakhstan University, Petropavlovsk 150000, Kazakhstan

**Keywords:** agro-industrial waste, cellulose extraction, lignocellulosic biomass, sustainable processing, biorefinery integration

## Abstract

The growing demand for sustainable and renewable materials has intensified interest in agro-industrial waste as an alternative source of cellulose. This review critically examines current approaches to cellulose production from major agro-industrial residues, including cereal straw, corn residues, rice waste, sugarcane bagasse, and oilseed by-products. Emphasis is placed on the relationship between feedstock composition and extraction efficiency, highlighting how lignin distribution, hemicellulose content, and mineral impurities influence pretreatment severity, cellulose yield, and process sustainability. The review systematically analyzes chemical, enzymatic, and mechanical processing routes, with particular attention being paid to pretreatment strategies, fibrillation intensity, and yield variability. Beyond cellulose recovery, key sustainability indicators—such as energy demand, water and chemical consumption, waste generation, and chemical recovery—are evaluated to provide a system-level perspective on process efficiency. The analysis demonstrates that cellulose yield alone is an insufficient criterion for sustainable process design and must be considered alongside environmental and techno-economic metrics. Advanced applications of agro-waste-derived cellulose are discussed using a feedstock-driven approach, showing that high functional performance can often be achieved with moderately processed cellulose tailored to specific end uses. Finally, the review addresses challenges related to feedstock heterogeneity, mineral management, standardization, and industrial scale-up, underscoring the importance of biorefinery integration, closed-loop resource management, and harmonized quality descriptors. These insights provide a foundation for the development of scalable and sustainable cellulose production pathways based on agro-industrial waste.

## 1. Introduction

The rapid growth of agro-industrial activities has led to the accumulation of large volumes of agricultural residues, including cereal straw, rice husk, sugarcane bagasse, and other lignocellulosic by-products. These materials represent an abundant and renewable biomass resource that is often underutilized or disposed of through open burning and landfilling, resulting in environmental pollution and loss of valuable carbon-based matter [1,2,3]. Among the main constituents of agro-industrial residues, cellulose is of particular interest due to its biodegradability, mechanical robustness, and broad applicability across industrial sectors, particularly in materials engineering and environmental applications [4].

In comparison with conventional wood-based feedstocks, agro-industrial residues exhibit pronounced compositional variability, higher ash content, and a heterogeneous distribution of cellulose, hemicelluloses, and lignin. These features complicate the extraction process and limit the direct transfer of established pulping and fractionation technologies originally developed for relatively homogeneous woody biomass [2,3]. Consequently, cellulose production from agro-industrial waste requires tailored processing strategies that carefully balance delignification efficiency, resource consumption, and environmental impact.

A wide range of chemical, physicochemical, and mechanical approaches has been reported for the isolation of cellulose from agricultural residues. While many studies demonstrate high extraction yields and effective lignin removal at the laboratory scale, such outcomes are frequently achieved through severe chemical conditions, extensive washing steps, or energy-intensive mechanical treatments [5,6]. As emphasized in recent reviews, cellulose yield alone is an insufficient indicator of process sustainability, as increased recovery is often accompanied by disproportionate consumption of energy, water, and chemical reagents [6,7].

Recent research has increasingly explored the conversion of agro-industrial biomass into micro- and nanostructured cellulose materials, driven by the enhanced surface area and functional properties of such products [8,9,10]. Numerous studies highlight the potential of nanocellulose derived from agricultural residues for advanced material applications, including composites, coatings, and functional films [2,11,12,13,14]. However, the production of micro- and nanocellulose typically involves additional mechanical or chemical processing steps that substantially increase energy demand and process complexity [5,6]. Therefore, the relevance of highly processed cellulose forms must be evaluated in the context of application-driven requirements and overall process efficiency, particularly when sustainability and scalability are considered. These sustainability trade-offs associated with additional chemical and mechanical processing are discussed in detail in Section 4.

Beyond extraction efficiency, the inherent structural features of cellulose play a crucial role in determining the performance of cellulose-based materials. Hydrogen bonding between cellulose chains is a primary factor governing stiffness and strength in solid cellulose assemblies, as demonstrated in studies on water-mediated hydrogen bond networks in cellulose nanofibers from wood aerogels [15]. Such molecular-level interactions influence not only mechanical properties but also process behavior during wet chemical and mechanical treatments, underscoring the importance of understanding cellulose structure when evaluating extraction strategies and downstream applications.

In this context, the present review critically examines sustainable approaches to cellulose production from agro-industrial waste, with a focus on feedstock characteristics, extraction technologies, yield variability, and process efficiency. Particular attention is devoted to sustainability-related aspects, including energy and water demand, chemical usage, waste generation, and challenges associated with scale-up and industrial implementation. By synthesizing recent advances and identifying key limitations, this review aims to provide a coherent framework for evaluating cellulose extraction routes from agricultural residues in terms of their environmental performance and industrial feasibility.

Although several review articles have addressed cellulose and nanocellulose extraction from agricultural biomass, most existing studies emphasize specific cellulose forms, laboratory-scale extraction routes, or application-driven performance metrics. In contrast, the present review adopts a feedstock-oriented and system-level approach, focusing on agro-industrial residues as heterogeneous and mineral-rich resources that impose distinct constraints on extraction efficiency, resource consumption, and waste management. By jointly analyzing feedstock composition, processing severity, yield variability, and sustainability indicators—including energy demand, water and chemical footprint, and side-stream management—this review provides an integrated framework for evaluating the industrial feasibility of cellulose production from agro-industrial waste.

## 2. Agro-Industrial Waste as a Source of Cellulose

Agro-industrial residues represent a widely available and low-cost source of cellulose. However, due to the structural complexity of plant biomass—especially the presence of lignin, hemicellulose, and silica—extracting high-purity cellulose requires careful selection of feedstock and optimized pretreatment protocols [16,17,18].

The main types of agro-industrial waste, such as oat and wheat straw, corn stalks, rice husks and sugar cane residues (bagasse), are characterized by a high content of polysaccharides [19], including cellulose (30–45%), hemicellulose (15–30%) and lignin (10–25%). This unique structure makes them promising feedstocks for processing into sustainable materials.

Using such waste has a double advantage: it reduces the environmental burden by utilizing biomass that would otherwise be burned or buried, and it creates a basis for the development of biomaterials using modern technologies. The inclusion of agro-industrial residues in production chains also supports the concept of a circular economy, which is becoming an important part of the global strategy for sustainable development [20].

Further consideration of their chemical composition and physicochemical properties allows for a deeper understanding of their potential as raw materials for the production of cellulose and its functionalization for various industries.

Each type of waste has its own chemical and physical characteristics that determine its suitability for various processing technologies. For example, waste with a high cellulose content (straw, bagasse) is suitable for the production of paper and biocomposites, while rice husks with a high silica content are used in sorbents and filtration materials [21].

Agro-industrial residues exhibit significant variation in their biochemical composition depending on crop type and region of origin. For instance, grain straw, including oat and wheat, has a high content of cellulose (30–40%) and hemicellulose (20–25%), as well as a balanced amount of lignin (15–20%) [22]. This makes it a universal raw material for the production of biocomposites, paper and biofuels. Corn straw has similar properties, but it has a lower lignin content (15–20%), which simplifies its processing and makes it promising for fermentation and the production of biodegradable materials [23]. In contrast, rice husk contains a high amount of ash-forming substances, primarily due to its elevated silica content. Rice husks present a unique challenge due to their high silica content (10–20%), alongside 30–35% cellulose [24]. This limits its use in the production of composites and paper, but makes it valuable for the creation of sorbents and filtration materials. Sugarcane (bagasse) is a fibrous material with a high cellulose content (40–45%) and a moderate proportion of lignin and hemicellulose (15–25%) [25]. Due to its structure, bagasse is widely used in the production of biodegradable packaging, paper, and energy products. Residues of oilseed crops, such as sunflower husks and soybean stalks, contain moderate levels of cellulose (35–40%) and are characterized by low ash content [26]. These residues often contain residual fatty substances, which makes them useful for the creation of sorbents and biofuels, but limits their use in other industries (Table 1).

Among the most versatile feedstocks are cereal straw and sugarcane bagasse, owing to their high cellulose content and balanced lignin levels. In contrast, residues such as rice husks and oilseed by-products exhibit specific compositional features—namely elevated silica content or residual lipids—that limit their use in conventional applications but create opportunities for more specialized functions, including sorbents and filtration media.

The pronounced differences in chemical composition, mineral content, and structural organization among agro-industrial residues necessitate a differentiated technological approach to cellulose recovery. Efficient valorization of these feedstocks relies on a sequence of processing steps, including pretreatment to disrupt the lignocellulosic matrix, cellulose extraction or hydrolysis to isolate the target polysaccharide, and, where required, fibrillation to obtain micro- or nanostructured cellulose. These processing strategies form the basis of the technologies discussed in the following section.

## 3. Technologies for Processing Agro-Industrial Waste into Cellulose

**Figure 1:**
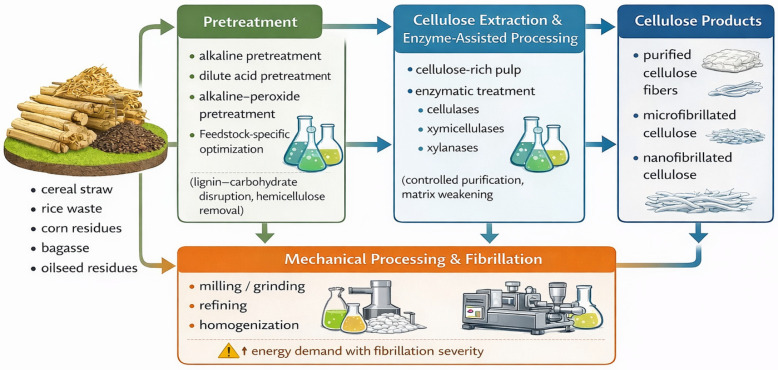
Schematic overview of the main technological stages involved in cellulose production from agro-industrial waste.

As shown in Section 2, agro-industrial residues such as cereal straw, corn residues, rice waste, sugarcane bagasse, and oilseed by-products exhibit pronounced differences in lignocellulosic composition, mineral content, and structural organization. These variations fundamentally determine the technological pathways required for efficient cellulose recovery. Consequently, cellulose production from agro-industrial waste is typically implemented through a sequence of interconnected stages, including pretreatment to disrupt the lignocellulosic matrix, cellulose extraction or hydrolysis to isolate the target polysaccharide, and, when necessary, mechanical processing to obtain micro- or nanostructured cellulose. This section discusses these technological stages with direct reference to the feedstocks introduced above and the corresponding processing strategies reported in the literature [27,28]. The main technological routes for cellulose production from agro-industrial waste are schematically illustrated in Figure 1.

Depending on the extraction severity and post-processing strategy, cellulose isolated from agro-industrial crops can be obtained in several common structural forms. Mild delignification and limited mechanical treatment typically yield cellulose fibers or lignocellulosic fibers retaining residual hemicellulose and lignin, which are widely used in packaging and composite applications. More intensive purification followed by controlled hydrolysis leads to microcrystalline cellulose, characterized by high crystallinity and reduced amorphous content. Additional mechanical or combined chemical–mechanical processing results in cellulose nanofibrils, consisting of long, flexible fibrillar networks, whereas strong acid hydrolysis produces cellulose nanocrystals with rod-like morphology and high stiffness. These structural forms represent the most common cellulose architectures reported for crop-derived biomass and form the basis for subsequent application-oriented processing.

### 3.1. Pretreatment Strategies for Lignocellulosic Disruption

Pretreatment governs the accessibility of cellulose within the lignocellulosic matrix and therefore represents the decisive stage in cellulose production from agro-industrial residues. Its primary function is not cellulose isolation itself, but the selective disruption of lignin–carbohydrate complexes and partial removal of hemicellulose, thereby enabling subsequent extraction or conversion steps. As a result, pretreatment efficiency directly influences both achievable cellulose yield and downstream processing [27,28].

For cereal straw (wheat and oat straw) and sugarcane bagasse, alkaline pretreatment is commonly employed to reduce hemicellulose content and weaken lignin–carbohydrate linkages, resulting in cellulose-enriched pulps suitable for further processing. In contrast, rice-derived residues require more carefully optimized pretreatment conditions because of their elevated mineral and silica content. Studies focusing on rice straw have demonstrated that alkaline and alkaline–peroxide treatments significantly modify the hemicellulosic fraction and improve cellulose accessibility by disrupting the rigid lignocellulosic structure [29]. Dilute acid pretreatment of rice straw has also been shown to enhance the efficiency of downstream enzymatic processing, provided that reaction severity is controlled to limit excessive cellulose depolymerization [30].

Corn residues are frequently subjected to dilute acid pretreatment to hydrolyze hemicellulose and increase porosity of the cell wall matrix. However, excessive acid severity may negatively affect cellulose integrity, highlighting the need for feedstock-specific optimization of pretreatment conditions [31]. Overall, pretreatment strategies must be tailored to the chemical and structural features of each residue to balance delignification efficiency, cellulose preservation, and process sustainability.

### 3.2. Cellulose Extraction and Enzyme-Assisted Processing

Cellulose extraction and enzymatic processing primarily govern the selectivity and controllability of lignocellulosic conversion rather than the initial disruption of biomass structure. These approaches are therefore most effective when applied as complementary steps following adequate pretreatment, particularly for agro-industrial residues in which residual hemicellulose and lignin fragments limit cellulose accessibility [28,30].

Enzyme-assisted processing relies on cellulases and hemicellulases to promote controlled hydrolysis of non-cellulosic polysaccharides while preserving the cellulose backbone. The efficiency of enzymatic treatments is strongly dependent on pretreatment-induced structural modifications, as enzyme penetration and activity are restricted in insufficiently disrupted biomass matrices. Accordingly, enzymatic routes function as process-intensification tools that enhance conversion efficiency but do not replace chemical or physicochemical pretreatment.

For corn stover, the role of enzyme composition has been investigated in detail. The incorporation of xylanases has been shown to enhance enzymatic hydrolysis by disrupting xylan–lignin associations and improving enzyme penetration into pretreated biomass, thereby increasing polysaccharide conversion efficiency [32]. More generally, xylanases are recognized as effective auxiliary enzymes that facilitate the removal of hemicellulosic barriers, supporting both hydrolysis and cellulose purification processes [33].

In the context of cellulose production from agro-industrial waste, enzymatic treatments should, therefore, be regarded as process-intensification tools rather than standalone alternatives to pretreatment. Their effectiveness relies on prior disruption of the lignocellulosic matrix and is strongly influenced by the structural characteristics of the selected feedstock. In addition to chemical and enzymatic pathways, physical size reduction and fibrillation represent an important complementary stage in cellulose processing, particularly when micro- or nanostructured materials are targeted.

### 3.3. Mechanical Processing and Fibrillation

Mechanical processing primarily governs fibrillation efficiency, particle size reduction, and energy consumption rather than delignification efficiency in cellulose production from agro-industrial residues. Accordingly, its role is auxiliary and strongly dependent on the effectiveness of prior chemical or enzymatic pretreatment, which determines the accessibility of cellulose within the lignocellulosic matrix.

Mechanical operations such as milling and grinding are typically applied either before chemical and enzymatic treatments to improve mass transfer, or after cellulose purification to induce fibrillation and generate micro- or nanostructured cellulose. These approaches are applicable across a wide range of feedstocks, including cereal straw, sugarcane bagasse, corn residues, and oilseed by-products [34].

While mechanical processing alone is insufficient to achieve effective delignification, it plays a critical role in controlling cellulose morphology and accessibility. Consequently, mechanical treatments are most effective when integrated within combined processing schemes involving chemical pretreatment and enzyme-assisted conversion, rather than being applied as standalone approaches.

### 3.4. Yield Variability and Comparative Considerations

Cellulose yields obtained from agro-industrial waste vary widely depending on biomass type, pretreatment severity, and extraction pathway. For instance, simple chemical extraction of cellulose from sugarcane bagasse has been reported at 18.6 ± 1.2% [21], whereas rice straw under similar conditions yielded only 7.8 ± 1.0% [35], indicating the influence of feedstock composition on recoverable cellulose content. Other studies also highlight that cereal straw and other fibrous residues generally provide more accessible cellulose fractions than mineral-rich residues such as rice waste, necessitating more intensive or optimized pretreatment to improve cellulose recovery [29,36]. Furthermore, differences in structural composition and degree of lignification between feedstocks affect cellulose accessibility and extraction efficiency.

Table 2 summarizes reported yields of purified cellulose obtained from selected agro-industrial residues following chemical or physicochemical processing. The reported values represent the fraction of dry biomass converted into cellulose-rich material after pretreatment and delignification.

From a sustainability perspective, reported cellulose yields represent not only a measure of process efficiency but also a key indicator of material and energy intensity. Higher cellulose recovery from fibrous residues such as cereal straw and sugarcane bagasse reduces the amount of chemical reagents, water, and energy required per unit of functional material produced. In contrast, mineral-rich residues such as rice husk often demand more intensive pretreatment to achieve comparable cellulose purity, which may negatively affect overall process sustainability if not properly optimized. Consequently, yield values should be interpreted in conjunction with pretreatment severity, chemical consumption, and downstream processing requirements rather than as isolated performance metrics.

Beyond sustainability considerations, the reported yields of purified cellulose also provide a quantitative basis for assessing the feasibility of subsequent nanocellulose production from agro-industrial residues. Owing to the complex lignocellulosic structure of raw biomass, nanocellulose cannot be produced directly from untreated feedstocks and instead requires multistep processing involving cellulose isolation followed by chemical, enzymatic, or mechanical conversion. As a result, the material efficiency of nanocellulose production relative to the initial biomass is constrained by both the yield of purified cellulose and the limitations of the applied processing routes, which together determine process scalability and techno-economic feasibility.

In this context, evaluation of available processing routes is essential to identify balanced strategies that reconcile yield, material quality, and process sustainability. Analysis of chemical, enzymatic, and mechanical processing routes indicates that no single method is universally optimal. Chemical pretreatments offer high efficiency and industrial scalability but generate chemical effluents; enzymatic approaches provide selectivity and milder processing conditions at the expense of longer treatment times; mechanical methods preserve cellulose integrity but are energy-intensive and insufficient as standalone processes. Consequently, integrated processing strategies that combine pretreatment, enzyme-assisted extraction or hydrolysis, and mechanical fibrillation are increasingly recognized as the most effective routes for producing cellulose and nanocellulose from agro-industrial waste with controlled yield and material characteristics.

Table 3 summarizes the key processing routes applied to agro-industrial residues, highlighting their objectives, applicability to different feedstocks, and principal advantages and limitations.

The comparison of chemical, enzymatic, and mechanical processing routes highlights that sustainability in cellulose production cannot be achieved through a single universal approach. Instead, sustainable process design requires balancing cellulose yield, chemical intensity, energy consumption, and scalability. While chemical pretreatments remain the most efficient and industrially mature options, their environmental footprint necessitates integration with reagent recovery, process intensification, or hybrid strategies. Enzymatic and mechanical methods, although inherently milder, are most effective when applied as complementary steps rather than standalone solutions. This interplay underscores the importance of integrated processing pathways for achieving both high material performance and environmental compatibility.

## 4. Sustainability and Process Efficiency in Cellulose Production

Sustainable cellulose production from agro-industrial waste should be regarded not merely as a technological task of polysaccharide isolation, but as an integrated engineering–environmental optimization of the entire processing chain, encompassing feedstock preparation, pretreatment, delignification and purification, and final material formation. In contrast to conventional pulp and paper industries—where technological schemes have evolved over decades around chemical recovery systems and internal energy self-sufficiency—the valorization of agro-industrial residues is still predominantly conducted using laboratory-scale or semi-pilot protocols. As a result, the most critical sustainability challenges are associated with high water and chemical demand, energy-intensive mechanical disintegration, and the absence of closed-loop reagent recovery.

Moreover, agro-industrial residues differ fundamentally from woody biomass due to their higher ash content, compositional variability, and often non-uniform structural organization, which directly affects process efficiency and environmental performance. Under these conditions, sustainability is determined by the combined effect of material, energy, and water consumption, the degree of process integration, and the management of side streams, including wastewater and solid residues. Therefore, a robust assessment of sustainability must rely on a multidimensional set of metrics, including cellulose yield and purity, specific consumption of energy, water, and chemicals, wastewater generation and the potential for reagent regeneration, as well as life-cycle considerations (LCA/LCI) that account for local energy infrastructure and system boundaries [41,42,43].

### 4.1. Material and Energy Efficiency of Cellulose Extraction

Material efficiency in cellulose production from agro-industrial residues is determined by the extent to which the initial lignocellulosic biomass can be converted into a cellulose-rich product while preserving a degree of polymerization and crystallinity sufficient for downstream applications [44]. However, a high cellulose yield alone does not necessarily imply process sustainability, as increased recovery is often achieved through harsher chemical conditions, higher reagent concentrations, elevated temperatures, and prolonged treatment times. These measures, while effective in promoting delignification, simultaneously increase chemical consumption, water usage, and energy demand. Therefore, a more appropriate assessment of material efficiency involves correlating cellulose yield with specific reagent consumption during delignification and bleaching (including washing stages) and with the subsequent energy profile of mechanical processing, when fibrillation is required [43].

**Figure 2:**
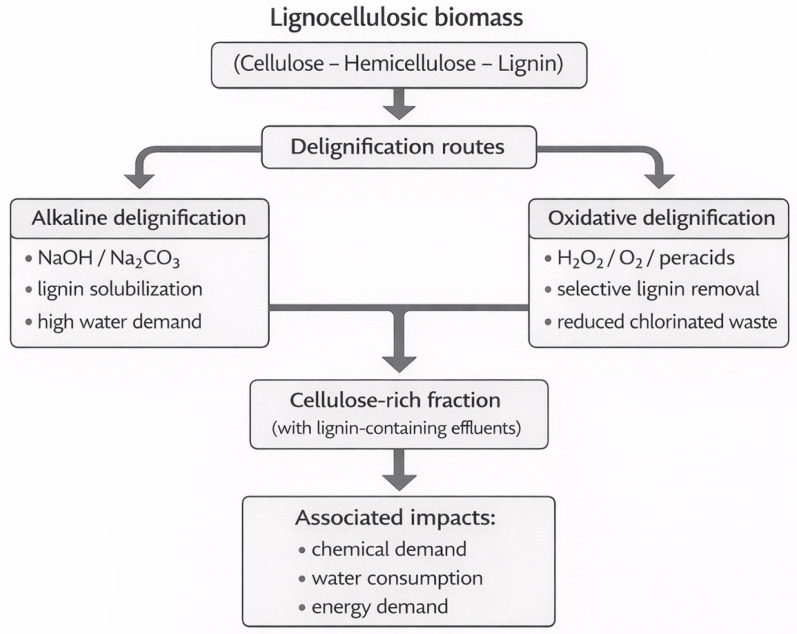
Main delignification routes applied to agro-industrial lignocellulosic biomass.

Agro-industrial lignocellulosic biomass consists of tightly bound cellulose, hemicellulose, and lignin fractions, which makes it inherently resistant to enzymatic and chemical hydrolysis without pretreatment [45]. Effective disruption of this structure is a prerequisite for cellulose extraction but is typically associated with significant material and energy inputs [44]. Conventional delignification routes rely on alkaline or oxidative reagents that efficiently cleave lignocellulosic linkages; however, they also generate a considerable chemical and water footprint and require substantial energy for heating, agitation, and post-treatment washing. The main delignification routes discussed above are schematically illustrated in Figure 2.

Energy demand becomes particularly critical when cellulose is processed beyond pulp-grade material into micro- or nanofibrillated forms. Reviews on cellulose nanofibers consistently emphasize that mechanical fibrillation techniques—such as high-pressure homogenization—lead to a non-linear increase in specific energy consumption with increasing pressure and number of passes. For example, reported values indicate an increase in energy demand from approximately 0.9 to 3.7 kWh·kg^−1^ as the number of homogenization cycles increases under constant pressure, illustrating the disproportionate energy penalty associated with achieving higher degrees of fibrillation [46]. These observations demonstrate that extensive mechanical disintegration can dominate the overall energy balance of the process [5].

From a sustainability perspective, these findings support the fit-for-purpose approach, whereby cellulose is processed only to the degree required for its intended application. For many large-scale uses—including packaging materials, structural biocomposites, filtration media, and sorbents—moderately purified or partially fibrillated cellulose may already provide sufficient functional performance, while avoiding the excessive energy demand associated with full micro- or nanofibrillation [5]. In this context, process intensification strategies, such as selective pretreatment and enzyme-assisted matrix weakening, are particularly valuable, as they reduce the mechanical energy required in downstream processing [43].

Life-cycle assessments further highlight that electricity consumption and chemical inputs are often the dominant contributors to climate change and resource depletion impacts in the production of highly processed cellulose materials. Importantly, these impacts are strongly influenced by the regional energy mix and the availability of renewable energy sources, making local energy infrastructure a critical factor in sustainability evaluation [41]. Recent studies on sustainable pretreatment strategies confirm that optimization of pretreatment severity—rather than maximal cellulose liberation—can significantly improve the overall environmental profile of cellulose extraction from agro-industrial waste [47,48,49]. Consequently, harmonized life-cycle inventories demonstrate substantial variability in environmental impacts across different processing routes, indicating that there is no universally optimal technology [41]. Instead, the most sustainable approach is feedstock-specific and context-dependent, determined by biomass composition, process configuration, and local energy and water systems.

### 4.2. Water and Chemical Footprint of Processing Routes

The water and chemical footprint of cellulose extraction from agro-industrial feedstocks is primarily governed by the need to remove lignin, hemicelluloses, and inorganic constituents from the lignocellulosic matrix. In comparison with woody biomass, agricultural residues often exhibit higher ash content and compositional heterogeneity, which intensifies washing requirements and complicates chemical recovery, particularly for mineral-rich feedstocks such as rice straw and rice husk. Consequently, water consumption and chemical usage become critical determinants of process sustainability [50].

Conventional processing routes based on alkaline pretreatment followed by oxidative bleaching remain highly effective in improving cellulose purity. However, these schemes generate strongly alkaline and/or oxidizing effluents and require substantial volumes of water for fiber washing and liquor neutralization. Chlorite- and hypochlorite-based bleaching systems, while efficient, are associated with elevated salt loads and increased toxicological risks in wastewater streams, motivating the search for alternative, environmentally benign oxidants [44].

In this context, a growing body of literature highlights the use of hydrogen peroxide–based systems as a comparatively greener alternative to chlorine-containing bleaching agents. Studies on cellulose and cellulose nanocrystal extraction from rice husk demonstrate that H_2_O_2_ can effectively promote delignification while reducing the formation of chlorinated by-products, although often at the expense of lower bleaching efficiency or stricter control of reaction conditions [51]. These findings indicate that reductions in chemical footprint are frequently achieved through process optimization and reagent substitution, rather than through the complete replacement of established delignification chemistries.

Beyond conventional aqueous systems, solvent-based pretreatment routes have been widely explored as tools for enhancing lignocellulosic disruption. In particular, ionic liquids (ILs) and deep eutectic solvents (DES) have demonstrated high efficiency in dissolving lignin and increasing cellulose accessibility. Nevertheless, comprehensive reviews emphasize that the sustainability of IL- and DES-based processes is almost entirely dependent on achieving high solvent recovery rates. The high cost of solvents, sensitivity to impurities, and gradual solvent degradation during recycling cycles represent major barriers to scale-up [52,53]. As a result, IL and DES systems should be regarded as process-intensification strategies rather than inherently green solutions, unless robust closed-loop solvent management is demonstrated.

Organosolv processes employing organic solvents such as ethanol or organic acids represent another promising approach, offering selective delignification and the possibility of solvent regeneration and reuse. When integrated into closed-cycle systems, organosolv routes can reduce both chemical and water footprints while enabling lignin valorization as a co-product. However, their overall sustainability remains strongly dependent on solvent recovery efficiency, energy demand for solvent separation, and potential impacts on cellulose quality [54]. A challenge specific to many agro-industrial residues is the elevated content of inorganic components, particularly silica in rice-derived feedstocks. High ash and silica levels interfere with alkali recovery, promote scaling and sludge formation, and increase water demand during washing operations. These effects hinder the closure of chemical loops and may substantially increase the water footprint of the process [55]. From an industrial perspective, this necessitates either prior demineralization of the biomass or the development of processing routes that are tolerant to high mineral content, underscoring the importance of feedstock-specific technology selection.

Overall, minimizing the water and chemical footprint of cellulose extraction from agro-industrial waste requires a balanced combination of moderate chemical severity, selective pretreatment strategies, and efficient recycling of process streams. Without such integration, improvements in cellulose purity and yield may be offset by disproportionate increases in water consumption and chemical waste generation.

### 4.3. Waste Streams, Effluents, and Biorefinery-Oriented Sustainability

In the context of agro-industrial cellulose extraction, environmental burden is largely defined by the management of process-derived waste streams rather than by the cellulose product itself. From an environmental standpoint, the dominant contributors to the overall burden of cellulose extraction from agro-industrial residues are the waste streams and effluents generated during processing, rather than the cellulose product itself. The most problematic flows typically include lignin-rich alkaline liquors after delignification, effluents from bleaching stages—particularly when chlorine-containing systems are employed—highly mineralized washing waters, and solid residues such as ash and sludge, which are especially pronounced in rice-derived feedstocks and other silica-rich agricultural wastes [56].

In conventional kraft pulping, these streams are effectively managed through integrated chemical recovery systems, in which spent liquors are concentrated and combusted, enabling regeneration of alkaline reagents (NaOH/Na_2_S) and simultaneous cogeneration of process energy. This recovery cycle is a key reason why mature pulp and paper mills can operate with relatively closed chemical and energy balances and comparatively low net waste discharge [57]. Although such infrastructure is rarely available in agro-based cellulose processing, the kraft process provides an important industrial benchmark for evaluating waste management strategies.

However, the direct transfer of kraft recovery logic to agro-industrial residues is often constrained by the elevated ash and inorganic content of agricultural biomass. High levels of silica and other mineral components interfere with alkali recovery, promote scaling and excessive sludge formation, and complicate effluent treatment, potentially increasing the environmental burden if conventional recovery schemes are applied without adaptation [58]. These limitations highlight the need for alternative strategies tailored to agro-based feedstocks.

As a result, the sustainability of cellulose production from agro-industrial waste is increasingly linked to biorefinery-oriented process integration, where cellulose is regarded as a core product within a broader valorization framework rather than as the sole output. In such systems, lignin-rich streams can be utilized for energy production or converted into value-added carbon-based materials and aromatic chemicals, while hemicellulosic fractions may serve as sources of fermentable sugars for biofuels and biochemicals. Mineral-rich residues, including silica-containing ash, can be further valorized in sorbents, fillers, or construction-related applications [59].

Beyond environmental considerations, this integrated biorefinery approach also improves economic resilience, as the co-production of multiple value streams enhances cost recovery from primary biomass processing. Several techno-economic and systems-level studies have demonstrated that waste valorization and by-product integration are essential for achieving industrially viable and scalable biomass conversion processes [60].

From a methodological perspective, reliable assessment of waste-related environmental impacts requires life-cycle assessment (LCA) frameworks with clearly defined system boundaries. Studies comparing cradle-to-gate and cradle-to-grave scenarios consistently show that conclusions regarding sustainability are highly sensitive to assumptions about effluent treatment, energy recovery, and by-product utilization. Consequently, reporting impact ranges rather than single-point values is recommended to capture the inherent variability of processing routes [61].

Overall, sustainable design of cellulose extraction processes from agro-industrial waste must simultaneously address three interrelated objectives:

- Reduction in chemically intensive and waste-generating steps;
- Integration of side-stream valorization within biorefinery concepts; and
- Implementation of waste and effluent management strategies that are compatible with the compositional characteristics of agricultural feedstocks.

Without such system-level integration, improvements in cellulose yield and purity may be offset by increased environmental burden associated with waste generation and treatment.

### 4.4. Integrative Considerations and Transition to Industrial Feasibility

Considering the sustainability aspects discussed above—including material efficiency, energy demand, water and chemical footprint, and waste management—it becomes evident that many laboratory-scale approaches for cellulose extraction from agro-industrial residues require substantial adaptation prior to industrial implementation. While high cellulose yields and effective delignification are frequently reported at the laboratory level, these outcomes are often achieved through increased chemical severity, extensive washing, or elevated energy input, which may limit scalability under industrial conditions [62].

**Figure 3:**
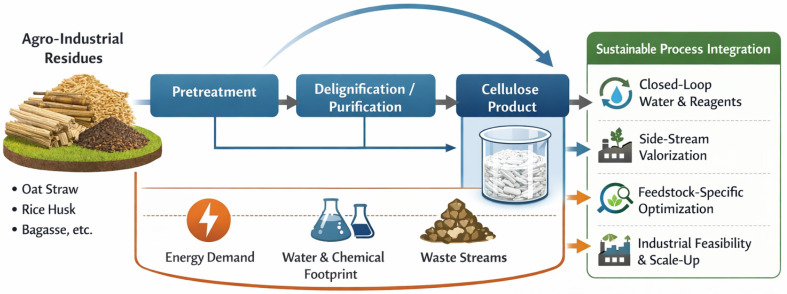
Integrated framework for sustainable cellulose extraction from agro-industrial waste.

A critical challenge in the transition toward industrial feasibility lies in the integration of individual unit operations into coherent and resource-efficient process chains. Industrial analyses consistently demonstrate that isolated optimization of pretreatment, bleaching, or mechanical processing stages is insufficient unless these steps are designed to operate within partially or fully closed cycles of water, reagents, and energy [63]. Without such integration, improvements achieved at the extraction stage may be offset by increased environmental burdens associated with effluent treatment, reagent losses, or energy-intensive downstream operations. The interdependence between extraction efficiency, resource consumption, and waste stream management is schematically summarized in Figure 3.

Figure 3 illustrates a conceptual framework for sustainable cellulose extraction from agro-industrial waste, emphasizing the balance between process severity and resource efficiency while accounting for feedstock-specific constraints, including compositional variability, elevated ash content, and the presence of mineral impurities. The scheme highlights that industrial feasibility is achieved not through maximal cellulose liberation at the laboratory scale, but through systems-level integration, encompassing closed-loop management of water and chemicals as well as the valorization of side streams generated during extraction.

For agro-industrial feedstocks, such system integration is particularly critical, as compositional heterogeneity and mineral content complicate process control and chemical recovery. Consequently, industrially viable cellulose extraction routes must be feedstock-specific, balancing pretreatment severity, product quality requirements, and the feasibility of reagent and water recycling. Recent reviews on agro-residue-based biomass processing consistently indicate that scalability depends on achieving robust and flexible operation under realistic and variable feedstock conditions rather than on maximizing cellulose yield alone [64].

These considerations demonstrate that sustainable cellulose production from agro-industrial waste cannot be assessed solely at the level of individual extraction steps. Instead, a systems-oriented perspective is required, in which extraction processes are evaluated in relation to their integration into broader material and energy management frameworks. This perspective provides the conceptual foundation for addressing industrial feasibility, scale-up challenges, and techno-economic constraints discussed in the following section [60].

## 5. Advanced Applications of Agro-Waste-Derived Cellulose

Agro-industrial residues are increasingly positioned not only as low-cost cellulose feedstocks, but as platforms for producing engineered cellulose forms (cellulose microfibers, microcrystalline cellulose, cellulose nanofibrils (CNFs), cellulose nanocrystals (CNCs)) that enable performance-driven materials for packaging, sorption, membranes, and biocomposites. Across applications, the most robust value propositions typically arise when

- the cellulose form is matched to the end-use (“fit-for-purpose” processing),
- the conversion route is tailored to feedstock-specific constraints (ash/silica for rice wastes, compositional variability for straws, extractives for oilseed residues), because these factors influence purification demand, fibrillation energy, and product reproducibility.

The Table 4 summarizes key types of cellulose materials derived from agro-industrial residues, their principal applications, and representative commercially available products or producers.

### 5.1. Oat and Wheat Straw-Derived Cellulose

Cellulose isolated from oat and wheat straw has been extensively investigated for applications requiring porous architectures and moderate mechanical reinforcement. Wheat-straw-derived cellulose nanofibers and lignocellulosic nanofibers have been successfully processed into lightweight aerogels exhibiting high adsorption capacities toward organic dyes, such as methylene blue, owing to their interconnected porous networks and accessible hydroxyl and carboxyl groups. These studies demonstrate that partial retention of lignin does not necessarily hinder adsorption performance, suggesting that highly severe delignification is not always required for environmental remediation applications [68,69].

Beyond adsorption, oat-straw-derived cellulose nanofibrils have been incorporated into starch-based biocomposites, resulting in enhanced tensile strength and reduced water vapor permeability. Such improvements are attributed to hydrogen bonding between cellulose fibrils and the polymer matrix, highlighting the role of fiber morphology and surface chemistry rather than extreme nanoscale refinement [70]. Additionally, functionalized lignocellulosic fractions from oat straw have been employed as fillers in elastomeric systems, where controlled carboxylation improved swelling behavior and mechanical stability, further broadening the application scope of cereal-straw cellulose [71].

### 5.2. Corn Residues-Derived Cellulose

Corn residues, including corn stover and corncobs, represent a major source of cellulose nanocrystals (CNCs) for high-performance film and coating applications. Corn-stover-derived CNCs have been widely used as reinforcing agents in poly(vinyl alcohol) (PVA) films, leading to substantial increases in tensile strength and Young’s modulus, as well as improved barrier properties against water vapor. These enhancements arise from the high crystallinity and aspect ratio of CNCs, which promote efficient stress transfer and densification of the polymer network [72].

Similarly, CNCs extracted from corncobs have been incorporated into PVA nanocomposites, where systematic studies demonstrate improved thermal stability and reduced permeability at relatively low CNC loadings. These findings confirm that corn-residue-derived cellulose is particularly well suited for advanced packaging and coating applications, where performance gains can be achieved without excessive material usage or processing complexity [73].

### 5.3. Rice Waste-Derived Cellulose

Rice straw and rice husk are distinctive agro-industrial residues due to their elevated mineral and silica content, which poses challenges during extraction but does not preclude advanced applications. Cellulose microfibers obtained from rice straw have been employed as reinforcement in thermoplastic starch films, yielding composites with improved mechanical strength and dimensional stability. These studies demonstrate that micro-scale cellulose structures can already provide sufficient reinforcement for packaging applications, avoiding the high energy demand associated with full nanofibrillation [74].

Rice-straw-derived cellulose has also been chemically modified and incorporated into PVA films, where enhanced interfacial interactions led to improved tensile performance and reduced moisture sensitivity [75]. In parallel, rice husk has been demonstrated as a viable precursor for CNCs with well-defined morphology and high crystallinity when alternative fractionation routes, such as formic/peroxyformic acid treatments, are applied. These CNCs are proposed as reinforcing elements in polymer nanocomposites, illustrating that even mineral-rich rice waste can be upgraded into high-value cellulose nanomaterials when processing routes are carefully designed [51].

### 5.4. Sugarcane Bagasse-Derived Cellulose

Sugarcane bagasse remains one of the most extensively studied agro-waste feedstocks for advanced cellulose applications. Cellulose nanofibrils (CNFs) derived from bagasse have been incorporated into starch-based films, resulting in significant improvements in tensile strength and reductions in water vapor permeability. Notably, several studies report that unbleached CNFs can perform comparably to bleached counterparts, suggesting that simplified processing routes may be sufficient for packaging-oriented applications [76].

Beyond starch matrices, bagasse-derived cellulose has been used as a reinforcing filler in polymer composites, where improvements in stiffness and mechanical stability have been observed. Additionally, CNCs isolated from bagasse have been incorporated into protein-based films, such as whey protein isolate matrices, leading to enhanced mechanical properties and barrier performance [77]. Bagasse cellulose has also been processed into aerogels with high porosity and oil-adsorption capacity, demonstrating its suitability for environmental cleanup and separation technologies [78].

### 5.5. Oilseed Residues-Derived Cellulose

Oilseed-related agro-residues, including rapeseed straw and soy hulls, are increasingly explored within integrated biorefinery concepts. Cellulose nanofibers isolated from rapeseed straw via chlorine-free processes have been applied as reinforcing agents in carboxymethyl cellulose films, yielding mechanically stable and biodegradable materials suitable for packaging and coating applications [79]. Complementary studies on rapeseed straw biorefineries emphasize the importance of co-product valorization, where cellulose extraction is integrated with the recovery of hemicellulose- and lignin-derived fractions [80]. Soy hulls represent a well-established oilseed residue for CNC production. CNCs derived from soy hulls have been used to reinforce natural rubber nanocomposites, where high aspect ratio and strong filler–matrix interactions resulted in marked mechanical reinforcement [81,82]. More recent studies further extend soy-hull-derived nanocellulose into hydrogel systems for food preservation, underscoring the versatility of oilseed-residue cellulose across multiple advanced material platforms [83].

## 6. Challenges, Standardization, and Scale-Up Outlook

Despite rapid progress in laboratory-scale protocols, the transition of agro-waste-derived cellulose extraction to industrially robust and environmentally competitive processes remains constrained by a set of recurring challenges: feedstock heterogeneity, mineral/ash-related process upsets, high utilities demand linked to separation and washing, and the need for verifiable standardization of product quality across batches. These limitations are especially pronounced for agro-industrial residues compared with wood, because seasonal and geographical variability in cellulose–hemicellulose–lignin ratios, extractives, and ash content directly affects pretreatment severity, delignification chemistry, and the feasibility of chemical recovery and water recycling. As a result, scale-up success depends less on maximizing cellulose liberation in a single “best” route and more on designing feedstock-specific, tolerant processing windows with clear quality targets and realistic recycling schemes.

A major bottleneck in scaling agro-based cellulose extraction is the management of mineral impurities, particularly silica-rich residues (e.g., rice husk and rice straw). High silica and ash levels can increase washing demand, generate inorganic sludges, and complicate alkaline recovery and downstream equipment operation (e.g., scaling and fouling), thereby weakening the sustainability gains expected from using waste biomass. In practice, industrially viable strategies increasingly align with zero-waste/valorization logic, where mineral-rich side streams are treated as co-products rather than disposal burdens. For example, sustainability-oriented recovery of silica (and associated nutrients) from rice-husk-derived ashes has been discussed as a route to reduce residual waste while improving overall resource efficiency, provided that recovery steps are integrated with process water management and residue handling [84].

Another decisive scale-up barrier is energy intensity and productivity in fibrillation or refining steps, when the targeted cellulose product requires high degrees of disintegration. Mechanical treatments (high-pressure homogenization, grinding, extrusion-based disintegration) are often the dominant energy sinks and can impose nonlinear penalties as processing intensity increases. Recent work quantifying energy–property trade-offs during homogenization under different processing consistencies illustrates that energy and cost efficiency are tightly coupled to how the mechanical step is engineered, reinforcing that “more fibrillation” is not automatically better from a sustainability or scale-up perspective [85]. In parallel, continuous-processing concepts—such as twin-screw extrusion routes enabling higher solids handling—have been advanced to improve throughput and reduce dilution-related utility demands, which is directly relevant for translating cellulose production from batch laboratory protocols to industrially meaningful mass balances [86].

From an industrial feasibility standpoint, a recurring conclusion across techno-economic and risk-informed analyses is that minimum product selling price, capital intensity, and operational risk are strongly sensitive to

- Solids content;
- Energy cost and electricity carbon intensity;
- Chemical recovery efficiency;
- Robustness to feedstock variability.

A financial and risk assessment of cellulose micro- and nanofibril manufacturing emphasized that productivity limits and cost structure are central obstacles to market adoption, which makes process intensification and stable quality control (not maximal disintegration) the key engineering priorities [87]. For agro-residues specifically, biorefinery-oriented demonstrations provide an instructive route: rapeseed straw processing has been shown in an integrated scheme where multiple fractions (e.g., hemicellulosic streams, cellulose-rich fibers, and lignin-rich liquors) are recovered, illustrating how co-product valorization can improve system-level sustainability and economics compared with single-product cellulose isolation [81].

A critical cross-cutting issue for scale-up is solvent and chemical-loop closure. Fractionation routes such as organosolv can yield comparatively clean cellulose fractions and high-quality lignin streams, yet large-scale feasibility is repeatedly linked to the energy and cost burden of solvent recovery and heat integration. A recent tutorial review on organosolv scale-up highlights that the main limitations are typically associated with solvent procurement and recycling energy demand, and that progress toward commercial operations depends on improved recovery design, robust operation across variable feedstocks (including straw/bagasse/wastes), and sustained techno-economic and life-cycle evaluation during piloting [88].

Alongside process engineering, standardization is becoming a prerequisite for credible scale-up and inter-study comparability. Even when the primary product is pulp-grade cellulose (not necessarily nanocellulose), industrial adoption requires consistent terminology, minimum specification sets, and transparent reporting of impurities (ash/silica), degree of polymerization, crystallinity-related indicators, and residual lignin/hemicellulose fractions. In the broader cellulose materials field, ISO has published terminology standards for cellulose nanomaterials, reflecting the general market need to reduce ambiguity and facilitate communication between researchers, manufacturers, and regulators—an approach that can be mirrored for agro-derived cellulose streams by adopting harmonized descriptors and reporting practices [89].

Finally, as production scales and occupational exposure scenarios become realistic, safety and risk considerations gain importance: dust formation during drying/handling, aerosol exposure during mechanical processing, and the behavior of fine cellulose fractions in the workplace environment. Reviews on pulmonary toxicity and hazard potential emphasize the need for standardized study designs, exposure characterization, and responsible risk management as cellulose materials move toward broader commercialization—issues that industrial feasibility roadmaps increasingly incorporate alongside Techno-Economic Analysis (TEA)/LCA [90,91].

Overall, the scale-up outlook for sustainable cellulose extraction from agro-industrial waste is most promising when processing is framed as a biorefinery-compatible, systems-optimized operation:

- Feedstock-specific severity control rather than “maximum extraction”;
- High-solids, high-throughput unit operations with quantified energy–property trade-offs;
- Demonstrably closed water/chemical loops (or clear recovery/valorization pathways for unavoidable side streams);
- standardized quality descriptors enabling reproducibility, procurement specifications, and regulatory confidence.

## 7. Conclusions

This review provides a comprehensive analysis of sustainable cellulose production from agro-industrial waste, emphasizing the influence of feedstock composition, extraction pathways, and system-level sustainability. Agro-residues such as cereal straw, corn residues, rice waste, sugarcane bagasse, and oilseed by-products represent viable alternatives to wood-based cellulose; however, their heterogeneous composition and variable mineral content necessitate feedstock-specific processing strategies. The results clearly demonstrate that cellulose yield alone is insufficient as a sustainability metric and must be evaluated alongside energy demand, water and chemical consumption, and waste generation.

Technological assessment shows that while chemical pretreatments remain the most effective routes for lignocellulosic disruption, their sustainability strongly depends on process severity, reagent recovery, and integration with mechanical or enzyme-assisted steps. Importantly, advanced applications of agro-derived cellulose indicate that high functional performance can often be achieved using moderately processed cellulose forms, supporting a fit-for-purpose approach rather than maximal fibrillation or purification.

Overall, the industrial feasibility of agro-waste-based cellulose production relies on systems-oriented design, including closed-loop water and chemical management, co-product valorization within biorefinery frameworks, and standardized quality descriptors. Addressing feedstock variability, energy-intensive processing stages, and scale-up constraints through integrated techno-economic and life-cycle perspectives will be essential for translating laboratory developments into commercially viable and sustainable cellulose-based materials.

## Figures and Tables

**Table 1:** Types of agro-industrial waste and their chemical composition.

Waste Type	Examples	Cellulose (%)	Hemicellulose (%)	Lignin (%)	Additional Components	Processing Methods	Potential Applications	Sources
Oat and wheat straw	Waste from grain crops after harvesting	30–40	20–25	15–20	Minerals (5–7%)	Acid hydrolysis, mechanical disaggregation	Biodegradable composites, biofuels, packaging	[22]
Corn Residues	Corn stalks, leaves, cobs	35–40	30–35	15–20	Extractive substances (5–10%)	Enzymatic hydrolysis, ultrasonic treatment	Biopolymers, filtration membranes	[23]
Rice Waste	Rice husks, rice straw	30–35	20–25	30–35	Silica (10–20%)	Alkaline treatment, mechanochemical destruction	Production of sorbents, composites, nanomaterials	[24]
Sugar Cane	Baggasse	40–45	15–25	20–25	Ash (2–5%)	Hydrothermal treatment, mechanical treatment	Biodegradable films, bioethanol	[25]
Oilseeds	Sunflower husks, soybean stalks	35–40	20–25	25–30	Oils (2–8%)	Alkaline treatment, steam treatment	Biocomposites, filters, carbon nanomaterials	[26]

**Table 2:** Yield of cellulose from agro-industrial waste.

Feedstock (Matches Section 2)	Product Reported in Study	Yield Definition Used by Authors	Reported Yield (%)	Key Processing Notes	Source
Wheat/oat straw (cereal straw)	Cellulosic pulp/cellulose-rich fraction	Pulp yield after alkaline pulping (mass basis reported by authors)	53–62	Soda–AQ pulping conditions; yield varies with severity	[37]
Corn residues (corncob = corn by-product)	Extracted cellulose	Yield (%) = weight of cellulose/weight of dried raw material × 100 (explicit formula)	38.18	Bleaching (acidified NaClO_2_) + alkaline extraction (KOH)	[36]
Rice waste (rice straw)	Extracted cellulose	Same explicit yield definition as above	32.26	Same bleaching + alkaline extraction route	[36]
Rice waste (rice husk)	Purified cellulose	Percentage of cellulose obtained after delignification and bleaching, reported relative to the initial mass of rice husk	52.3	Alkaline delignification with 12% NaOH at 80 °C for 3 h followed by bleaching using 2.5% NaOCl at 80 °C for 1 h; washing to neutral pH	[38]
Sugarcane residues (bagasse)	Pulp/cellulosic fraction	Reported pulp yield from bagasse processing (as reported in the cited study)	39.59	Bagasse-to-cellulose route summarized with stated yield	[39]

**Table 3:** Comparison of chemical, enzymatic, and mechanical processing routes for cellulose production from agro-industrial waste.

Processing Route	Main Objective	Applicable Agro-Industrial Residues	Key Features	Advantages	Limitations	Sources
Chemical pretreatment (alkaline, dilute acid, alkaline–peroxide)	Disruption of lignocellulosic matrix; removal of hemicellulose and partial delignification	Wheat and oat straw; corn residues; rice straw; sugarcane bagasse; oilseed residues	Solubilization of hemicellulose; weakening of lignin–carbohydrate complexes; increased cellulose accessibility	High efficiency; well-established; suitable for scale-up	Chemical consumption; wastewater generation; risk of cellulose degradation at high severity	[28,30,40]
Enzyme-assisted processing (cellulases, hemicellulases, xylanases)	Enhancement of polysaccharide conversion and cellulose accessibility after pretreatment	Corn residues; rice straw; cereal straw	Selective hydrolysis of polysaccharides; effectiveness strongly dependent on pretreatment and enzyme composition	Mild conditions; high selectivity; reduced chemical load	High enzyme cost; long processing times; ineffective without pretreatment	[28,30,32,33]
Mechanical processing (milling, grinding, refining)	Particle size reduction and cellulose fibrillation	Wheat and oat straw; sugarcane bagasse; corn residues; oilseed residues	Increased surface area; improved mass transfer; fibrillation to micro-/nanostructured cellulose	No chemical reagents; preservation of cellulose structure	High energy demand; no delignification when used alone	[34]

**Table 4:** Advanced applications of cellulose derived from agro-industrial waste and representative commercial products.

Cellulose Type	Agro-Waste Source	Key Applications	Representative Commercial Examples/Producers
Nanocellulose/cellulose pulp	Rice straw, sugarcane bagasse, fruit peels	Sustainable packaging materials, personal care, biocomposites	Cellupro Green: Cellulose Pulp @ PC, EcoPulp@CP (agro-waste derived) [65]
Cellulose nanocrystals (CNC)	Corn residues, other biomass	Reinforcement in coatings, packaging, composites	CelluForce (CelluRods®)—commercial CNC supplier [66]
Cellulose nanofibrils (CNF)	Various agro-biomass	Barrier films, biocomposites	Producers such as Borregaard AS, Stora Enso Biomaterials (market nanocellulose) [65,67]

## Data Availability

No new data were created or analyzed in this study.
